# Graphene-Induced Hyperthermia (GIHT) Combined With Radiotherapy Fosters Immunogenic Cell Death

**DOI:** 10.3389/fonc.2021.664615

**Published:** 2021-08-16

**Authors:** Malgorzata J. Podolska, Xiaomei Shan, Christina Janko, Rabah Boukherroub, Udo S. Gaipl, Sabine Szunerits, Benjamin Frey, Luis E. Muñoz

**Affiliations:** ^1^Department of Internal Medicine 3 - Rheumatology and Immunology, Friedrich-Alexander-University of Erlangen-Nürnberg (FAU), Universitätsklinikum Erlangen, Erlangen, Germany; ^2^Deutsches Zentrum für Immuntherapie (DZI), Friedrich-Alexander-University Erlangen-Nürnberg and Universitätsklinikum Erlangen, Erlangen, Germany; ^3^Department of Otorhinolaryngology, Head and Neck Surgery, Section of Experimental Oncology and Nanomedicine (SEON), Else Kröner-Fresenius-Stiftung Professorship, Universitätsklinikum Erlangen, Erlangen, Germany; ^4^Univ. Lille, CNRS, Centrale Lille, Univ. Polytechnique Hauts-de-France, UMR 8520-IEMN, Lille, France; ^5^Translational Radiobiology, Department of Radiation Oncology, Universitätsklinikum Erlangen, Friedrich-Alexander-Universität Erlangen-Nürnberg (FAU), Erlangen, Germany

**Keywords:** reduced graphene oxide, immunogenic cell death, hyperthermia, multimodal, radiotherapy, melanoma

## Abstract

Radiotherapy and chemotherapy are the standard interventions for cancer patients, although cancer cells often develop radio- and/or chemoresistance. Hyperthermia reduces tumor resistance and induces immune responses resulting in a better prognosis. We have previously described a method to induce tumor cell death by local hyperthermia employing pegylated reduced graphene oxide nanosheets and near infrared light (graphene-induced hyperthermia, GIHT). The spatiotemporal exposure/release of heat shock proteins (HSP), high group mobility box 1 protein (HMGB1), and adenosine triphosphate (ATP) are reported key inducers of immunogenic cell death (ICD). We hypothesize that GIHT decisively contributes to induce ICD in irradiated melanoma B16F10 cells, especially in combination with radiotherapy. Therefore, we investigated the immunogenicity of GIHT alone or in combination with radiotherapy in melanoma B16F10 cells. Tumor cell death *in vitro* revealed features of apoptosis that is progressing fast into secondary necrosis. Both HSP70 and HMGB1/DNA complexes were detected 18 hours post GIHT treatment, whereas the simultaneous release of ATP and HMGB1/DNA was observed only 24 hours post combined treatment. We further confirmed the adjuvant potential of these released DAMPs by immunization/challenge experiments. The inoculation of supernatants of cells exposed to sole GIHT resulted in tumor growth at the site of inoculation. The immunization with cells exposed to sole radiotherapy rather fostered the growth of secondary tumors *in vivo*. Contrarily, a discreet reduction of secondary tumor volumes was observed in mice immunized with a single dose of cells and supernatants treated with the combination of GIHT and irradiation. We propose the simultaneous release of several DAMPs as a potential mechanism fostering anti-tumor immunity against previously irradiated cancer cells.

## Introduction

Every anti-tumor therapy aims to induce immunogenic cell death (ICD), which favors the development of specific anti-tumor responses. The spatiotemporal exposure of calreticulin on the outer leaflet of the plasma membrane (1, 2), the secretion of ATP (3, 4), and the release of DAMPs such as HMGB1 (4–6), heat shock protein 70 (HSP70) (7, 8) and HSP90 (4, 9) are essential organic adjuvants required to induce ICD. These signals are recognized by various pattern recognition receptors on antigen presenting cells facilitating their activation and migration to draining lymph nodes followed by induction of potent adaptive immune response (10). The presence of one of the organic adjuvants is not sufficient to induce proper immune reactions and must be accompanied by additional signals. We have postulated that the release from dead cells of both ‘find-me’ (ATP) and danger signals (HMGB1 and HSP90) is enough to support robust immune responses, whereas when only one of the adjuvants concurs, anti-tumor immunity fails (4). Some mediators released by dying cells, such as Prostaglandin E2 or adenosine, show immunosuppressive features contributing to the tolerance (11, 12) and growth of tumor cells (13).

Besides sensitizing tumor cells to radio- and chemotherapy (14), hyperthermia has been demonstrated to have a direct cell killing effect (apoptosis or necrosis) in both *in vitro* and *in vivo* conditions (15–17). This is achieved by the denaturation and aggregation of intracellular proteins that are not seen in the case of radio- or chemotherapies (18–22). Temperatures above 44°C cause extensive cell damage due to sudden protein aggregation and result in necrosis, whereas apoptosis is usually elicited in the case of moderate hyperthermia (i.e., 41.5°C) (23, 24). There are hints that the mode of action inducing the heat has a decisive effect on the cell death (25). Nuclear proteins and components of the Mre11-Rad50-Nbs1 complex orchestrating the repair of double strand breaks in DNA are the most prone to heat-induced degradation (26–29). Hence, the energy dose (temperature) and time jointly orchestrate the systemic outcome. This means that the generation and control of the heat are essential parameters to be modulated. With the lack of instruments assuring homogenous heat dispersion, profound damage can be induced to the surrounding tissues. The prevention of the latter and targeting invisible metastasis are the main challenges of this field and are still under development. Although shrinking tumors, sole hyperthermia cannot substitute any actual therapy (30). Nevertheless, hyperthermia is undoubtedly sensitizing tumor cells for further treatments (25, 31–33).

Gamma irradiation induces irreversible double-strand DNA breaks leading to apoptotic cell death. Dying tumor cells *in vivo* are sensed by the immune system propagating predominantly tolerogenic messages (34–36). Whether hyperthermia complementing radiotherapy results in ICD has not been investigated in-depth yet. We have recently shown that PEGylated reduced graphene oxide nanosheets (rGO-PEG) are biocompatible, non-toxic, and can be used for intravenous application to induce fine-tuned localized hyperthermia by application of near infrared radiation (37). We demonstrate herein that tumor cells killed by the combination of gamma irradiation and hyperthermia release several DAMPs in a fashion that renders dead B16F10 melanoma cells immunogenic.

## Materials and Methods

### Gamma Irradiation (X rays)

B16F10 melanoma cells derived from the C57BL/6 mouse (ATCC, #CRL-6475) were exposed to ionizing irradiation (20 Gy, 120 kV, 22.7 mA; GE Inspection Technologies, Germany).

### Graphene-Induced Hyperthermia (GIHT)

B16F10 melanoma cells were exposed to GIHT as described before (37). The cells were seeded in 24-well flat-bottom culture plates (2 × 10^5^ cells/well). Next, graphene nanosheets (50 µg/ml) were placed in transwell inserts (0.4 µm pores) in close proximity to the cells, and plates were exposed to near-infrared irradiation (NIR, 960 nm, 1 hour, 2 W/cm^2^) applied by Hydrosun^®^750 (Hydrosun Medizintechnik, Müllheim, Germany). The lower compartment’s temperature was registered every 10 s with a Voltcraft K204 Thermometer (Voltcraft, Wollerau, Switzerland) and a high sensitive “in-well” temperature probe.

### Flow Cytometric Analysis of Cell Death

The supernatants (SNs) containing detached B16F10 melanoma cells treated with X-ray irradiation, GIHT, or a combination of both were collected 24 hours post treatment into polypropylene tubes. Remaining adherent cells were exposed to trypsin-EDTA solution for 5 min at room temperature (RT), and detached cells were added to their corresponding SN fractions. Cells kept at 37°C and 5% CO_2_ served as control of cell death and normal cell turnover. Harvested cells were centrifuged at 300×g for 5 min, and a morpho-physiological characterization of cell death by flow cytometry measurement was performed as described before (38). Briefly, the cells were resuspended in a four-color staining solution containing 1 µg/ml of Annexin A5 (AxA5)-FITC (ImmunoTools, Friesoythe, Germany), 100 ng/ml of PI (Sigma-Aldrich, Taufkirchen, Germany), 10 nM 1,1′,3,3,3′,3′-hexamethylindodicarbo - cyanine iodide (DiIc1(5), Enzo Life Sciences, Lörrach, Germany), 1 μg/ml of Hoechst 33342 (Thermo Fisher Scientific Inc., Waltham, USA) in Ringer’s solution for 30 min at RT followed by acquisition on Gallios flow cytometer and analysis with the software Kaluza 2.1.

### Detection of Danger Signals

Plates containing treated B16F10 melanoma cells and specified controls were centrifuged at 300xg for 5 min at the indicated time points, and the SNs were collected. The release of ATP from B16F10 melanoma cells was detected with the ‘Luminescent ATP Detection Assay Kit’ (Abcam, Cambridge, UK). ATP degradation was prevented by the provided lysis buffer. Luminescence measurements were performed on a Centro LB960 luminometer. HMGB1 and HSP70 were detected with the HMGB1 ELISA Kit II (IBL International, Hamburg, Germany) and DuoSet IC Kit (R&D Systems (Minneapolis, USA), respectively, according to the manufacturer’s instructions. For the measurement of absorbance, an ELISA Microplate Reader and the software Magellan 7.1 SP1 were used.

### Splenocytes Isolation and Staining

Briefly, Balb/c mice were sacrificed, and dissected spleens were pressed through a 70 µm cell strainer washed with ice-cold PBS. Collected cells were centrifuged at 300xg for 5 min at 4°C. Erythrocytes were lysed with erythrocytes lysis buffer for 2 min, followed by centrifugation at 300xg for 5 min at 4°C. Splenocytes proliferation was detected with the CellTrace™ CFSE Cell Proliferation Kit (Thermo Fisher Scientific, Rockford, USA) employed according to the manufacturer’s instructions. In brief, splenocytes (10e6 cells/ml) were incubated in 5 µM staining solution for 20 min at RT. Excessive dye was removed by adding the medium with 10% serum for 5 min at RT. Next, labeled cells were centrifuged at 300xg for 5 min and were employed in further experiments.

### Dendritic Cell Generation and Activation

Femora and tibia bones from sacrificed C57BL/6 mice were sterilized in 70% ethanol. Next, bone marrow was washed out with a needle (0.4 mm x 19 mm) into ice-cold medium. Collected cells were filtered through a 70 µm cell strainer and centrifuged at 300xg for 5 min at 4°C. Bone marrow-derived cells were differentiated with a complete cell culture medium containing 4 ng/ml of GM-CSF (ImmunoTools, Friesoythe, Germany) and 10 ng/ml IL-4 (ImmunoTools, Friesoythe, Germany) for 7 days. On days 3 and 5, a fresh DCs medium was added. DC cultures were treated with 100 µl of SNs from B16F10 melanoma cells treated with X-ray irradiation, GIHT, or a combination of both for 24 hours at 37°C and 5% CO_2_. The expression of co-stimulatory molecules on DCs was confirmed after the conditioning treatment by flow cytometry using the following antibodies anti-mouse MHC II (1:600, Biolegend, San Diego, USA), anti-mouse CD11c (1:800, Biolegend, San Diego, USA), CD40 (1:800, Biolegend, San Diego, USA), CD86 (1:400, Biolegend, San Diego, USA).

### T Cell Activation and Proliferation

Conditioned DCs were irradiated (20 Gy) and co-incubated with CFSE-stained splenocytes for four days, at 37°C and 5% CO_2_. After 4 days splenocytes were stained with anti-mouse CD3 (1:400, Thermo Fisher Scientific, Rockford, USA), anti-mouse CD4 (1:600, Biolegend, San Diego, USA), and anti-mouse CD8 (1:800, Biolegend, San Diego, USA) antibodies added for 30 min at RT in the dark and analyzed with Gallios flow cytometer and the software Kaluza 2.1. The mean fluorescence intensity (MFI) of T cells exposed to unprimed DCs was used as the maximal signal to calculate the dilution of the dye induced by proliferation. The average number of divisions (division index) was obtained by dividing the maximal MFI signal by the signal obtained from T cells exposed to DCs pre-incubated with the indicated conditions.

### Mice

All mice experiments were conducted in full agreement with institutional guidelines on animal welfare and with the approval of the local Animal Care and Use Committees of the University Erlangen-Nürnberg and the ‘Regierung von Unterfranken’ [Allowance numbers TS-12/2015 (bone marrow cells and splenocytes); 55.2 DMS-2532-2-103 (airpouch model); 54-2532.1-6/12 (tumor growth)].

### Air-Pouch Model

Briefly, 5 mL of sterile air was injected subcutaneously in the back of previously anesthetized mice (isofluorane). The air formed a cavity between the skin and the fascia of the back of the thorax. This cavity was stabilized with 3 ml of sterile air after three days. After five days the cellular membrane formed allows the study and quantification of infiltrating leukocytes. On day five, 5 mL of supernatants collected 24 hours post-treatment from B16F10 melanoma cells treated with X-ray irradiation, GIHT, or a combination of both were injected into airpouches. After 24 hours, the mice were sacrificed, and the lavage of pouches was collected. Lavages were centrifuged for 5 min at 300x g and stained for 30 min at room temperature in the dark with the following antibodies: α-ms CD45 (Biolegend, San Diego, USA), α-ms CCR3 (Biolegend, San Diego, USA), α-ms CD11b (Thermo Fisher Scientific, Rockford, USA), α-ms Ly-6C (Biolegend, San Diego, USA), α-ms Ly-6G (Biolegend, San Diego, USA), α-ms CD170 (Siglec-F) (Biolegend, San Diego, USA), α-ms CD115 (Biolegend, San Diego, USA), α-ms F4/80 (Biolegend, San Diego, USA). Fluorescence was measured on a Gallios cytofluorometer, and data analysis was performed with the software Kaluza 2.1 Following populations were distinguished: inflammatory monocytes (CD45pos CD11bpos Ly6Chigh Ly6Gneg CCR3neg SiglecFneg), anti-inflammatory monocytes (CD45pos CD11bpos Ly6Clow Ly6Gneg CCR3neg SiglecFneg), macrophages (CD45pos CD11bpos CD115pos F4/80pos), neutrophils (CD45pos CD11bpos Ly6Cpos Ly6Gpos CCR3neg SiglecFneg).

### Evaluation of the Efficiency of the Killing Method

In order to determine whether supernatants of treated cells contained surviving cells that might preclude their use as immunization agent, supernatants containing detached dead and dying cells were transferred to a new culture flask containing fresh DMEM supplemented with 10% (v/v) FBS and penicillin–streptomycin and cultured at 37°C in a 5% CO_2_ atmosphere. Cell survival and ability to form colonies were investigated 7 days post-transfer. Microphotographs were taken on Microscope Axiovert 25 by a Nikon D700 reflex camera. Images were processed using Adobe Photoshop CS5. Also C57BL/6 mice were injected intraperitoneally (i.p.) with supernatants of treated cells. Mice were sacrificed once tumor growth in the peritoneal cavity was detected by simple inspection and palpation. The experiment ended at 32 days, and surviving mice were sacrificed. Results are presented as Kaplan-Meier survival curves (**Figure 2**).

### Anti-Tumor Immunization

A syngeneic anti-tumor immunization model was used. Mice (C57BL/6, MHC haplotype H2b) were immunized i.p. with supernatants containing detached dead and dying cells harvested 24 hours post-treatment from B16F10 melanoma cells (carriers of the MHC haplotype H2b). SNs from cells treated with gamma irradiation or the combination of GIHT and gamma irradiation were used in this experiment. GIHT alone was not used as immunization since the inoculum contained surviving cells and was not suitable as an immunization agent (**Figure 2**). After 14 days, the mice were challenged subcutaneously (s.c.) in the back with viable B16F10 melanoma cells (1 × 10^6^). The width, height, and depth of subcutaneous tumors were measured with a caliper and recorded for a maximum 16 days.

### Statistics

Statistical analysis was performed by GraphPad Prism (version 7.0) software. As statistically significant, the p-values ≤ 0.05 were considered.

## Results

### GIHT Triggers Apoptosis Rapidly Followed by Secondary Necrosis

**Figure 1:**
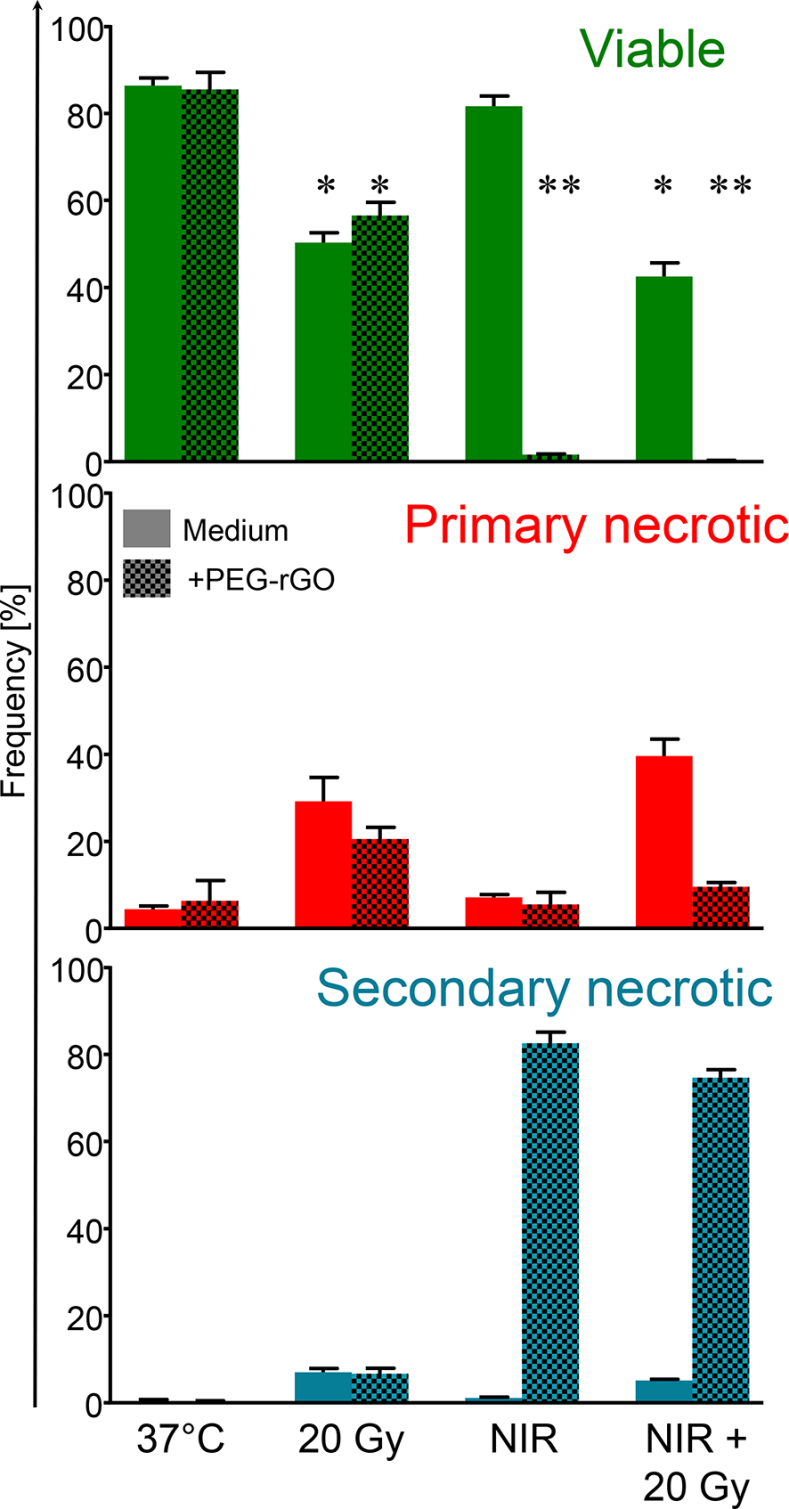
Frequencies of subpopulations of dead and dying cells. B16F10 melanoma cells exposed to gamma irradiation (20 Gy), graphene induced hyperthermia (GITH) or the combination were classified by flow cytometry employing multiparametric cell staining after 24 hours. Viable cells, green, negative for PI and AxA5. Primary necrotic cells, red, positive for PI and AxA5 with high DNA content (Hoechst). Secondary necrotic cells, blue, positive for PI and AxA5 with low DNA content (Hoechst). NIR, near infrared irradiation; Gy, Gray; PI, propidium iodide; AxA5, annexin A5. *p < 0.05; **p < 0.01.

Anti-tumor therapies induce various types of cell death that might result either in the activation or in the inhibition of specific anti-tumor immune responses. For example, the survival of cancer patients has been negatively correlated with tolerogenic apoptosis (39), and primary necrosis was shown to lack of immunogenicity (4). Therefore, we first evaluated the type of cell death induced by GIHT, gamma irradiation and its combination *in vitro* employing a flow cytometry-based six-parameter classification protocol (**Supplementary Figures 1, 2**) (38). Untreated cells display a high proportion of viable cells (**Figure 1**). The exposure of B16F10 melanoma cells to gamma irradiation alone results mainly in primary necrosis independently of GIHT (**Figure 1**). NIR exposure caused hyperthermia and rapid progression to secondary necrosis when rGO or rGO-PEG were present (GIHT, **Supplementary Figure 2**) (37). This phenotype persisted after the combined action of gamma-irradiation and GIHT (**Figure 1**).

### Surviving Cells Are Present in Supernatants of Dead and Dying Cells

**Figure 2:**
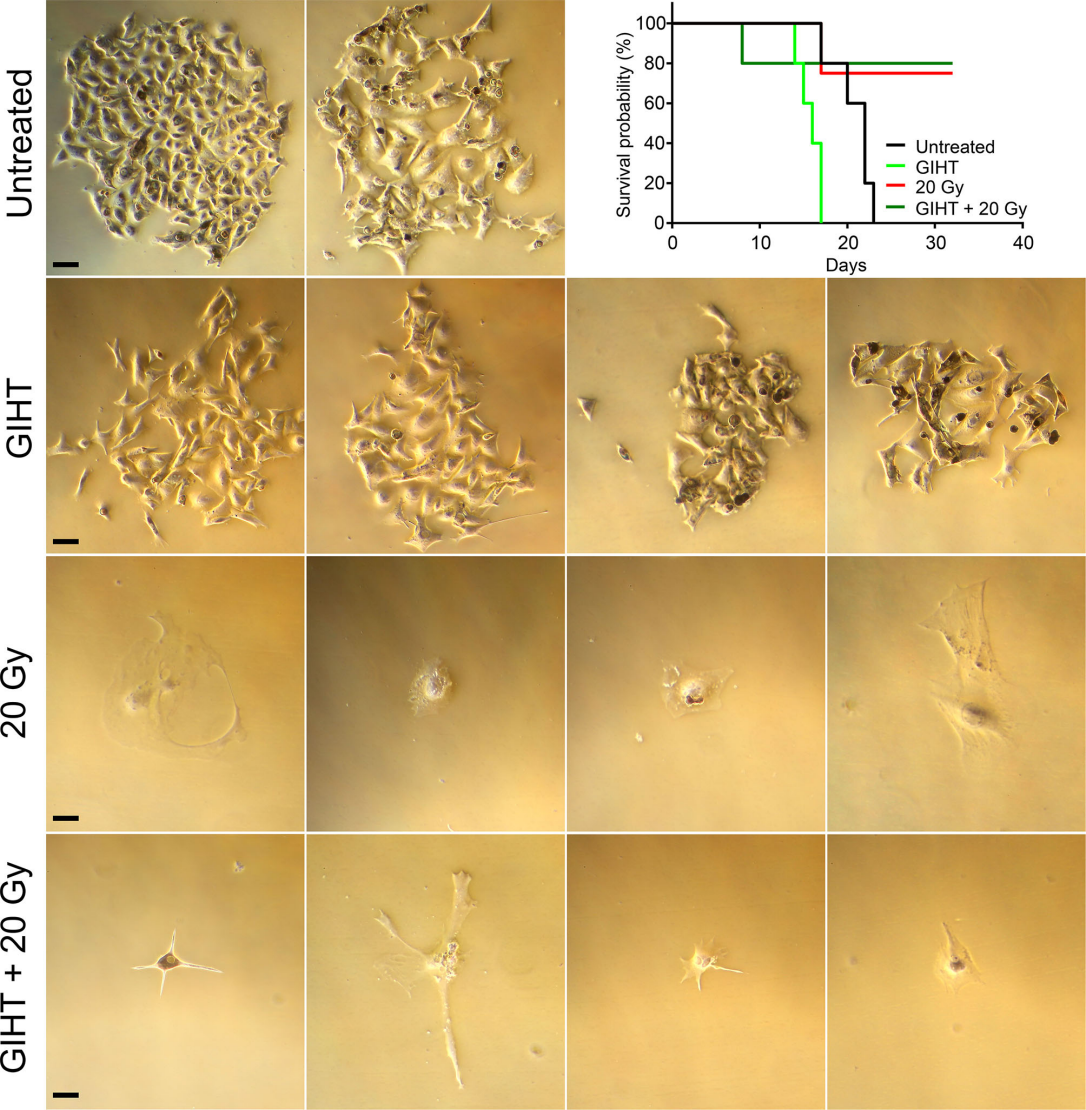
Efficiency of cell killing. B16F10 melanoma cells exposed to gamma irradiation (20 Gy), GIHT or the combination. Supernatants containing detached dead and viable cells were collected 24 hours post-treatment and cultured for further 7 days adding fresh medium twice. Representative bright field pictures of cultures show growth of colonies after 7 days of treatment. Supernatants were also injected in the peritoneal cavity of C57Bl/6 mice. Mice were observed during 32 days or euthanized before if the growing tumor compromised their wellbeing. Kaplan-Meier survival analysis of mice (n=4 or 5) treated with the indicated supernatants. Scale bar, 25 µm. Gy, Gray; GIHT, graphene induced hyperthermia; i.p. intraperitoneal.

The stimulation of proliferation of few surviving cells by bystander dead cells has been confirmed for melanoma cells, fibroblasts, and primary synoviocytes (13) and it might contribute considerably to relapses after radio- or chemotherapy (40–42). In order to determine the suitability of dying tumor cells supernatants as immunization adjuvants, we further cultured supernatants containing detached dead and dying cells in culture flasks and in the peritoneal cavity of C57Bl/6 mice. The supernatants of untreated cells and those treated with GIHT contained surviving cells that generated colonies after 7 days of cultures *in vitro* and tumors in the peritoneal cavity of mice, respectively (**Figure 2**). Contrarily, supernatants of cells treated with gamma irradiation and with the combination of irradiation and GIHT did not generate colonies *in vitro* or peritoneal tumors *in vivo* (**Figure 2**). This indicates that killing of B16F10 melanoma cells by hyperthermia alone might cause the release of growth and survival factors that support the growth of tumors at the site of injection of supernatants. This precludes the use of cells treated with hyperthermia alone in immunization protocols.

### Dying Cells Killed by GIHT Combined With Gamma Irradiation Induce Inflammatory Cell Infiltration in the Site of Injection

**Figure 3:**
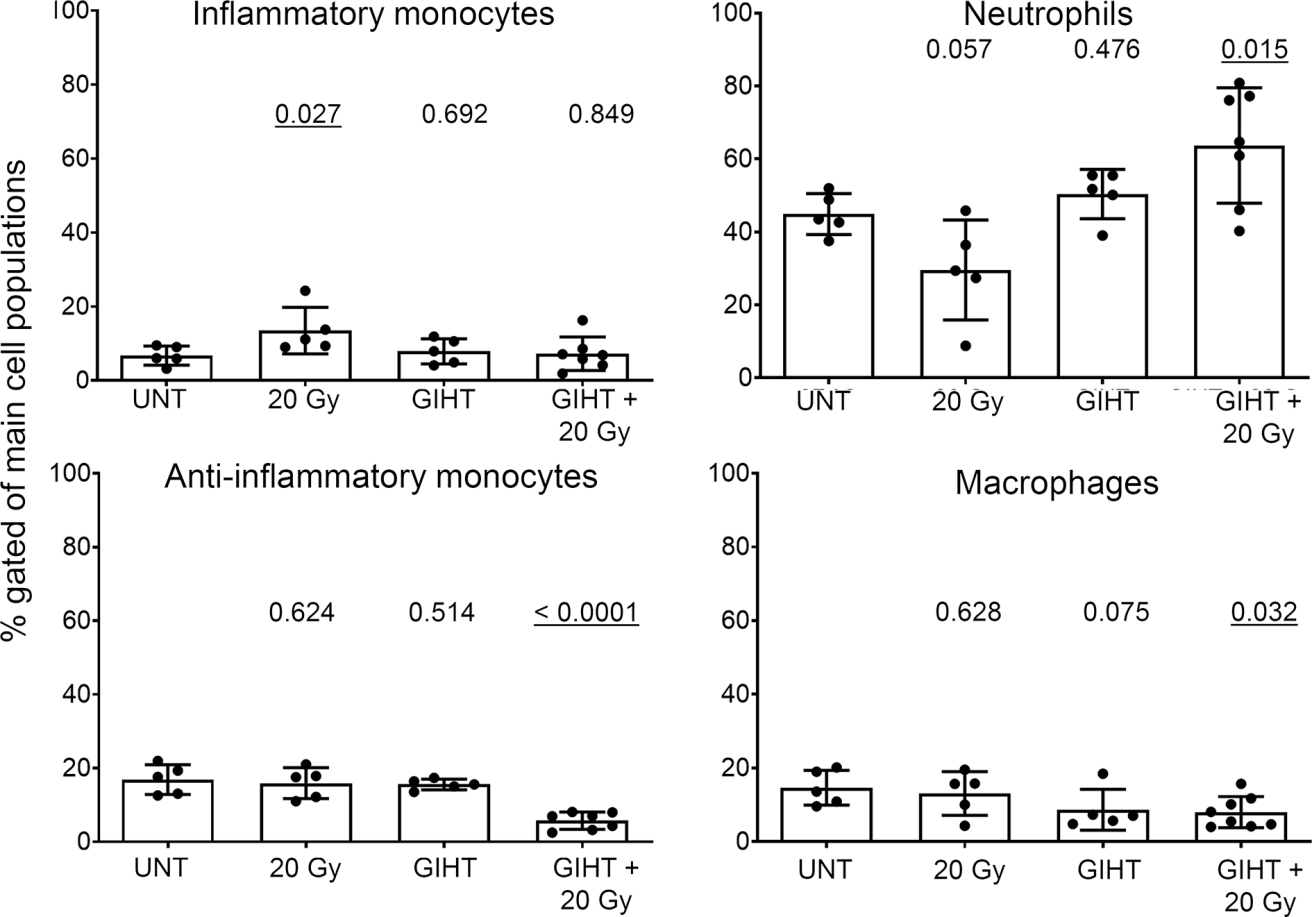
Inflammatory cell infiltration to the site of cell death. SNs of dying B16F10 melanoma cells (24h) were injected into air pouches of mice and the infiltrating cells were quantified by flow cytometry. The infiltration caused by supernatants of untreated cells was used as a baseline. The main four myeloid populations are shown. One-way analysis of variances of five mice with Tukey’s multiple comparison test is shown. Values of p < 0.05 considered as significant are underlined. UNT, untreated; Gy, Gray; GIHT, graphene-induced hyperthermia.

Employing the *in vivo* airpouch model, we investigated the pro-inflammatory potential of mediators released by dead cells induced by GIHT alone or in the combination with gamma irradiation (**Figure 3**). Supernatants of dead and dying cells were injected into established sterile airpouches. We observed a significant increase in the infiltration of inflammatory neutrophils into airpouches supernatants induced by the combination of therapies accompanied by a significantly decreased proportion of anti-inflammatory monocytes and macrophages. Supernatants of irradiated cells caused a moderate elevation of inflammatory monocytes (**Figure 3**).

### GIHT Combined With Gamma Irradiation Elicits the Release of Organic Adjuvants With a Specific Spatiotemporal Pattern

**Figure 4:**
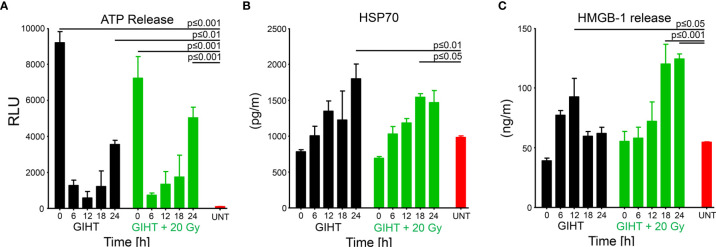
Organic adjuvants released from GIHT treated B16F10 melanoma cells. **(A)** Time kinetic of the levels of ATP, **(B)** HSP70, and **(C)** HMGB-1 detected in supernatants after the application of the treatment. Supernatants from untreated cells (UNT) represent basal concentrations of DAMPs. The two-way analyses of variance with Bonferonni posttest was employed. Values of p < 0.05 were considered as significant. Means with the standard error of the mean (SEM) are shown. GIHT, graphene induced hyperthermia; UNT, untreated; ATP, adenosine triphosphate; HSP70, heat shock protein 70; HMGB1, high mobility group box 1 protein.

We further analyzed the presence of organic adjuvants released by dead and dying B16F10 melanoma cells after GIHT (37) (**Figure 4**). We observed an early (t0) and late (t24) release of ATP in the case of GIHT applied alone or in combination with gamma irradiation (**Figure 4A**). Also, both treatments induced late release of HSP-70 (**Figure 4B**). However, only the combination of GIHT and 20 Gy was associated with a late secretion of HMGB-1 (**Figure 4C**), suggesting the release of nucleosome-bound HMGB-1 as reported for secondary necrotic cells (43).

### Dying Cells Treated With GIHT and X-Rays Induce the Proliferation of Naive T Cells *In Vitro*

**Figure 5:**
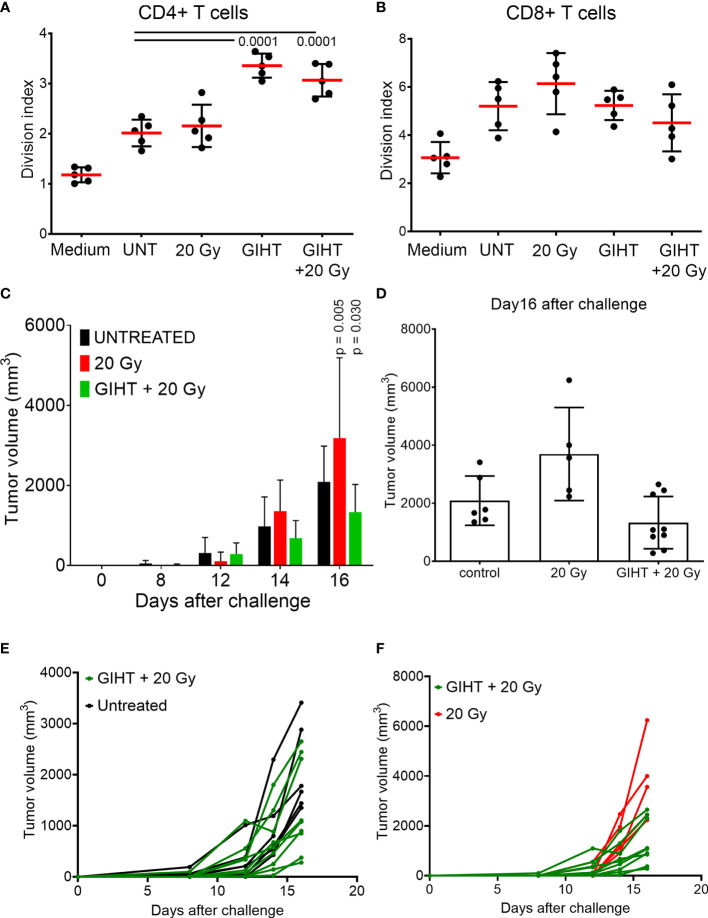
Induction of specific anti-tumor immune responses. T cells proliferation induced by conditioned DCs. DCs were co-incubated for 24 hours with SN collected from B16F10 melanoma cells exposed to indicated treatments. CSFE-labelled T cells were co-cultured with DCs for 4 days. To show bottom-line proliferation, DCs were conditioned with fresh medium. Division index of CD4 positive T cells **(A)** and CD8 positive T cells **(B)** is shown. Kruskal-Wallis test with Dunn’s multiple comparison test was employed. Values of p < 0.05 were considered as significant. **(C)** Tumor growth after challenge of immunized mice. Immunization was performed with a single i.p. dosis of detached B16F10 melanoma cells including their supernantants. Fourteen days after the immunization, mice were challenged with viable B16F10 melanoma cells injected subcutaneously in the back. Two-way ANOVA was used to compare the means at each time point. Values of p < 0.05 after Tukey’s multiple comparison test were considered as significant and depicted. **(D)** Tumor volume of immunized mice at day16 after the challenge with viable B16F10 melanoma cells. One way ANOVA was applied to evaluate the means at day 16 after challenge. **(E**, **F)** Tumor growth of single mice of the experiment shown in **(C)**. P values of Fisher´s least significant differences are depicted. Gy, Gray; GIHT, graphene-induced hyperthermia.

After confirming the presence of released organic adjuvants and testing its inflammatory potential, we aimed to investigate whether these adjuvants contribute to the activation of DCs. The supernatants of all treatments caused a significant upregulation of the activation markers CD80, CD86, MHC-II and CD40 on bone marrow derived DCs (**Supplementary Figure 3**). These conditionally activated DCs were used in a modified mixed lymphocyte reaction (MLR) to activate naive allogeneic T cells to proliferate (**Figures 5A, B**). The allogeneic major histocompatibility complex (MHC) molecules induced so-called background stimulation of T cells proliferation (**Figures 5A, B**, UNT). We observed that the proliferation of CD4+ T cells but not CD8+ T cells were significantly increased in response to SN from tumor cells exposed to GIHT alone or in combination with gamma irradiation (**Figures 5A, B**).

### Dying Cells Killed by GIHT Combined With Gamma Irradiation Elicit Specific Anti-Tumor Immune Responses *In Vivo*

Once we observed that innate and adaptive immune activation was induced by the SN from tumor cells killed by the combination of hyperthermia and gamma irradiation, we sought to determine whether these SN are able to support specific anti-tumor responses if inoculated together with dead tumor cells in an immunization/challenge experiment. For this experiment we used the SN from untreated (containing no viable tumor cells), gamma irradiated alone (containing dead tumor cells) and in combination with GITH (containing dead tumor cells) as a single immunization dose. Mice were challenged with viable tumor cells after 14 days, and tumor growth was monitored for 32 days. The SN of cells exposed to the single treatment modality of gamma irradiation fostered the growth of secondary tumors while the combination of gamma irradiation and GITH resulted in a discreet reduction of the tumor volume of secondary tumors (**Figures 5C–F** and **Supplementary Table 1**).

## Discussion

Radiotherapy is an essential treatment option for the majority of patients bearing tumors (44). However, radioresistance of some cancer cells results in the failure of this therapy (45). Hyperthermia was demonstrated to radiosensitize tumor cells (46, 47). The effect of GIHT administered before radiation on the progression of cell death of the poorly immunogenic B16F10 melanoma cells was investigated. Twenty Gy resemble the two weeks cumulative dose of X-rays that patients receive when undergo radiotherapy (31).

We have observed that rGO and rGO-PEG exhibit the best photothermal conversion efficacy (37). The hyperthermia (42-43°C) induced by rGO and rGO-PEG alone or in combination with gamma irradiation led to significantly increased cell death. When GIHT was administered alone or in combination with gamma irradiation, melanoma cells mainly followed apoptotic cell death patterns with fast progression to secondary necrosis. The decision taken by the dying cell is orchestrated by multiple factors, such as the severity of the damage, energy availability, the presence/absence of ligands of cell death/dependent receptors, or inhibitors of specific pathways. The outcome has profound effects on the subsequent immune response (48). Necrotic cell death does not always induce robust immune responses (4), and the activation of apoptosis might result as a double edge sword with features of immunogenic (4, 49, 50) or tolerogenic (36) (40) cellular demise. Necroptotic cells, for example, although releasing ‘find me’ signals, may be engulfed without activating the immune system (51). Therefore, determining the precise death pathway and delineating its immunological consequences results of major importance while designing novel anti-cancer therapies.

When apoptotic cells are not cleared in an efficient and timely manner, they become secondary necrotic (52). *In vivo*, the complete apoptotic program’s execution is usually interfered by rapid phagocytosis (53). However, in the case of large amounts of cell demise that challenges the capacity of phagocytes to efficiently clear cellular debris (54) or when the clearance capacity is itself reduced (35, 55), apoptotic cells lose their plasma membrane integrity and release immune stimulators (56, 57). Secondary necrosis *in vivo* is linked to multiple inflammatory and autoimmune disorders (35, 58, 59). Based on our observations, we suggest that when GIHT is applied in combination with gamma irradiation, a large number of apoptotically modified tumor-derived antigens along with an appropriate cocktail of mediators are released and can stimulate DCs. This is possible due to the high frequency of secondarily necrotic cells observed with the multimodal therapy.

The efficiency of anti-cancer therapies rely on many factors. One of them is the microenvironment resulting directly after therapy. Massive cell death of solid tumors changes dramatically the tumor microenvironment and triggers biological reactions in the host and tumor. Inosine released by dead and dying cells mediates proliferation of surviving cells *via* purinergic receptors (13) and this might support the appearance of relapses (40, 42). The treatment with GITH alone was inefficient and fostered the rapid proliferation of surviving cells in our *in vitro* and *in vivo* settings.

It was demonstrated that the spatiotemporal appearance of organic adjuvants such as ATP (3, 4), HMGB1 (4–6), HSP70 (7, 8, 60, 61) and HSP90 (4, 9) decides about the consequences of cell demise. In line with this, we previously proposed that the sole presence of ATP leads to the silent removal of dead cells, whereas the presence of ATP together with HMGB1 or HSP90 induces the robust anti-tumor immune responses (4).

We detected significantly increased ATP release levels into the extracellular space mostly due to a temporal heat-induced permeability of membranes (62, 63), including mitochondrial envelopes. Notably, the recovery of plasma membranes occurs in the latest 40 minutes after the treatment since most of the cells are PI negative by this time point. The progression towards secondary necrosis was then responsible for the releases of further intracellular contents later on (24 hours).

We detected significantly increased secretion of HSP70 at 24 and 12 hours post administration of GIHT alone or combined with gamma irradiation, respectively. Exposure to elevated temperatures leads to the increased expression of intracellular HSP. Colorectal adenocarcinoma cells exposed to 41.5°C for 1-hour show significantly decreased cell death orchestrated probably by the Thermo protection effect of HSP (64). In terms of ICD, when HSP proteins are presented on the plasma membrane’s outer leaflet or are released in the extracellular milieu, they gain immune stimulatory properties (7, 8, 60, 61). In other studies, hyperthermia (41.5°C, 1 hour) administered alone or in combination with radiation (2 Gy) was demonstrated to trigger the release of both proteins HSP70 and HMGB1 by dead and dying B16F10 melanoma cells (65). HSP70 secreted after the treatment elicits the maturation of DC and promotes the release of pro-inflammatory cytokines (64).

Chronic persistent inflammation is linked to tumorigenesis, and extracellular HMGB1 is perceived as a pro-inflammatory cytokine inducing the expression of other inflammatory factors (66–68). Besides that, HMGB1 leads to the secretion of other pro-inflammatory cytokines (i.e., TNF, IL-1, or IL-6) by resident or migrated leukocytes (69). In this manner, HMGB1 further fosters a vicious cycle of inflammation and manipulation of the immune system. The plethora of actions of HMGB1 can be explained by its redox status, the type of affected cell, and available receptors (70), as well as by its interaction with DNA. Free reduced HMGB1 protein was shown to be passively released by primary necrotic cells, whereas the oxidized form, which is additionally bound to the nucleosome, was observed in secondary necrotic cells (43, 71). It was reported that during apoptosis, cysteine residues of HMGB1 are oxidized by mitochondrial ROS produced in a caspase-dependent manner. This fosters immunological tolerance. Immunogenicity was then recovered by blocking its oxidation (72). Furthermore, apoptotic cell death accompanied by elevated intracellular levels of ROS exhibited higher immunogenicity *in vivo* when compared to the death developing in the absence of ROS (71).

In the death induced by GIHT alone, the release of danger signals was significantly increased at the earlier time points (HMGB1/DNA, 12 hours) when ATP was still absent. In the case of the combined treatments, we detected significant concentrations of extracellular DAMPs, HSP70, and HMGB1/DNA, 18 hours post treatment. The combinational therapy was then characterized by the simultaneous increase of ATP and HMGB1/DNA released 24 hours post treatment. We have previously observed that dying cells are less potent stimulators when ATP is released without other DAMPs (4). The presence of only one of the organic adjuvants is not enough to provide sufficient stimulation of the immune system. Werthmöller et al. reported that the simultaneous presence of HSP70 and HMGB1 was linked to the increased immunogenic potential of cellular demise (65). This suggests that the DAMPs detected 18 hours post combinational treatment can stimulate the immune system. The additional presence of ATP further fosters the activation.

At the site of inoculation of supernatants, we observed significant infiltration of neutrophils and significantly decreased levels of anti-inflammatory monocytes in response to cells treated with GIHT combined with gamma irradiation. Single treatments affected less the composition of early infiltrates. It was demonstrated that dying cancer cells secrete specific chemokines to recruit cells of the immune system (11, 73). IL-1α and IL-1β were reported to attract neutrophils (initial phase) and macrophages (late phase) during sterile inflammation, respectively (74). Additionally, IL-1α was shown to sustain chronic infiltration of neutrophils (75). After migration to the site of inflammation, exposure of neutrophils to ‘eat me’ signals, such as PS and calreticulin, results in the polarization to pro-inflammatory phenotype and, consequently, to cytotoxicity towards remaining cancer cells that survived the therapy (11). Therefore, we speculate that the observed infiltration of neutrophils might further support our multimodal therapy’s anti-tumor potential.

Finally, we demonstrated that cell death induced by GIHT alone or in combination with gamma irradiation resulted in the activation of DCs, which stimulated the proliferation of CD4+ T cells *in vitro*. This suggests that released ATP and HMGB1-DNA complexes are potent supporters of T cell activation. Employing dead and dying cells in an immunization/challenge experiment, we observed a decreased tumor volume in the group of mice immunized with the combined treatment. Contrarily, gamma irradiation alone fails to induce protection against tumor growth. We suggest that the significant release of several DAMPs significantly contributes to increased immunogenicity of B16F10 melanoma cells. Therefore, GIHT could be implemented in multimodal therapies since it may take advantage of radio sensitization of tumor cells by inducing the timely release of ATP and HMGB1-DNA complexes during the progress of cell death.

The melanoma B16F10 clone implanted in immunocompetent syngeneic mice allows the study of tumor growth preserving the interactions between cancer cells and the microenvironment (76–78). However, this clone has the disadvantage to have a high proliferative and metastasizing capability (79) that precludes the use of viable cells for immunization. Therefore, the antigenic load at the immunization site might be insufficient to trigger immune responses that resulted in tumor free mice after the challenge. Nevertheless, our observations are significant enough to propose the study of the principle of radiosensitation using nanosheets-targeted hyperthermia in other solid tumors models in future research. When biocompatible rGO-PEG nanosheets are applied intravenously, they become enriched in well-vascularized tumors by the enhanced permeability and retention effect. These nanosheets can be then stimulated with deep penetrating NIR irradiation to achieve fine-tuned localized hyperthermia (GITH) in solid tumors.

## Data Availability Statement

The raw data supporting the conclusions of this article will be made available by the authors, without undue reservation.

## Ethics Statement

The animal study was reviewed and approved by Animal Care and Use Committees of the University Erlangen-Nürnberg and the ‘Regierung von Unterfranken’.

## Author Contributions

Conceptualization, MP, CJ, BF, SS, and LM. Methodology, MP, XS, CJ, BF, and RB. Software, MP, XS, and CJ. Validation, MP, CJ, SS, and LM. Formal analysis, MP, XS, CJ, BF, UG, and LM. Investigation, MP, CJ, BF, and LM. Resources, RB, UG, SS, and LM. Data curation, MP, BF, UG, RB, CJ, and LM. Writing and original draft preparation, MP and LM. Writing—review and editing, MP, BF, UG, RB, SS, CJ, and LM. Visualization, MP, BF, CJ, and LM. Supervision, BF, SS, and LM. Funding acquisition, BF, SS, and LM. All authors contributed to the article and approved the submitted version.

## Funding

This research was funded by the EU through the Marie Sklodowska-Curie action (H2020-MSCA-RISE-2015, PANG-690836) and by the Manfred-Roth-Stiftung, Fürth, Germany.

## Conflict of Interest

The authors declare that the research was conducted in the absence of any commercial or financial relationships that could be construed as a potential conflict of interest.

## Publisher’s Note

All claims expressed in this article are solely those of the authors and do not necessarily represent those of their affiliated organizations, or those of the publisher, the editors and the reviewers. Any product that may be evaluated in this article, or claim that may be made by its manufacturer, is not guaranteed or endorsed by the publisher.
