# Supplementary material for: Graphene-Induced Hyperthermia (GIHT) Combined With Radiotherapy Fosters Immunogenic Cell Death
Source: Front Oncol. 2021 Aug 16;11:664615. doi: 10.3389/fonc.2021.664615 (PMC8415397; doi:10.3389/fonc.2021.664615)
Supplement: Supplementary file 1 [file DataSheet_1.pdf]

Supplementary figure 1.

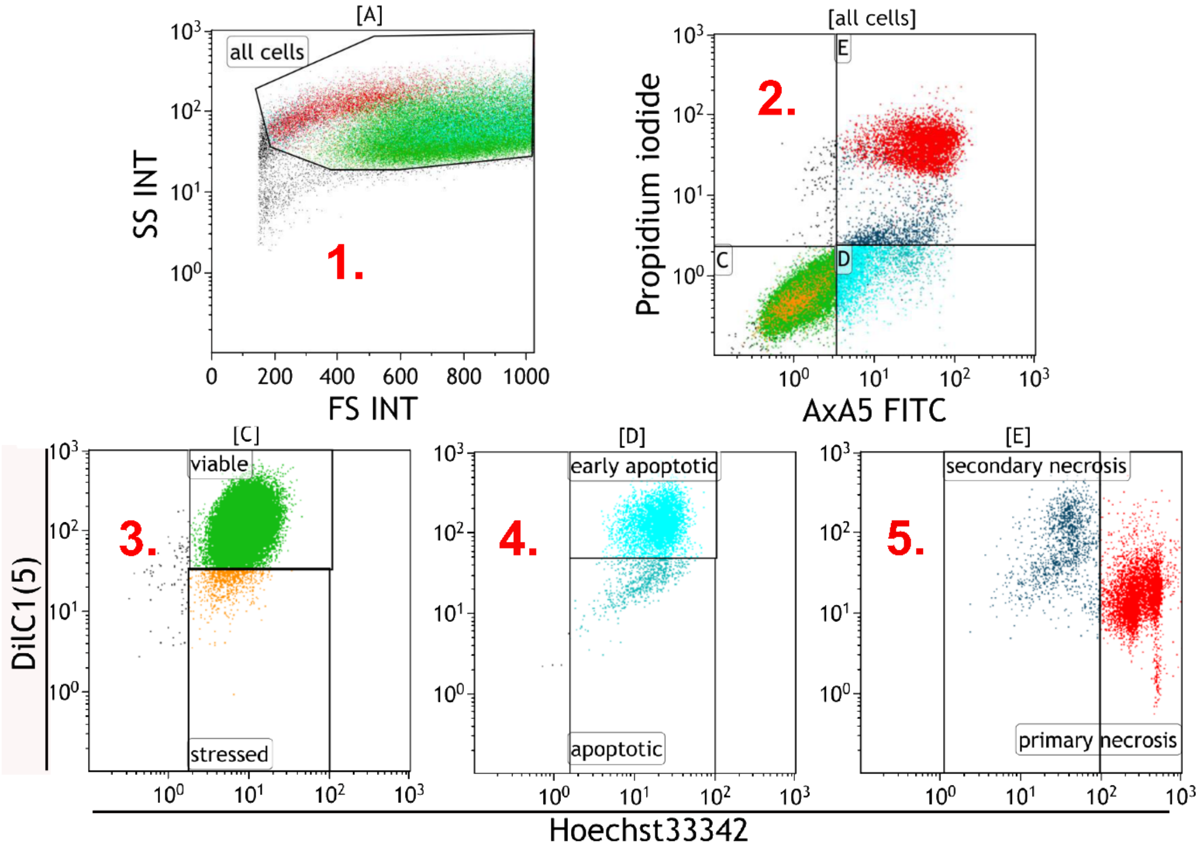

Gating strategy for multiparametric characterization of cell death. Representative plots of 20 Gy exposed cells. Viable and dead cells were gated (all cells) independently of their granularity (SS) and size (FS) (1). Next, the fluorescence of PI and Annexin A5 was plotted (2). Cells negative for PI and AxA5 were further classified into viable or stressed cells according to their mitochondrial potential (Dilc1(5), high or low, respectively) (3). Cells positive for AxA5 and negative for PI were further gated into early apoptotic cells and apoptotic cells according to their mitochondrial potential (Dilc1(5), high or low, respectively) (4). Cells positive for both, PI and AxA5, were classified into primary necrotic cells and secondary necrotic cells depending on their DNA content (Hoechst, high or low, respectively) (5). PI, Propidium iodide; AxA5, annexin A5; Gy, Gray.

Supplementary figure 2.

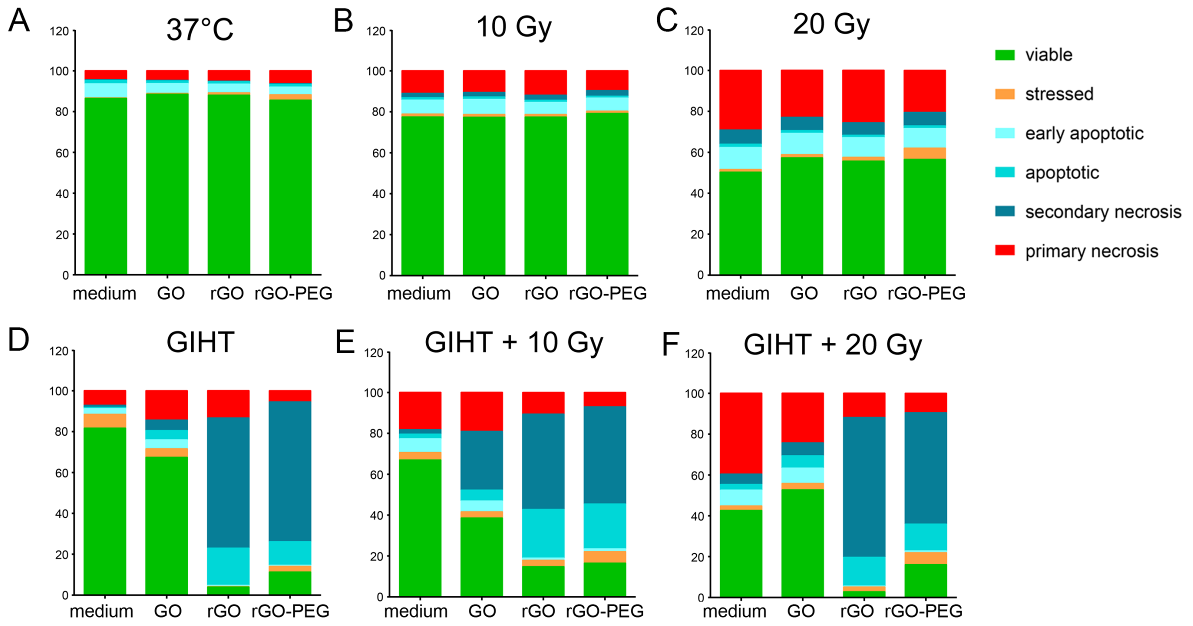

Frequencies of subpopulations of dead and dying cells. B16F10 melanoma cells were exposed to the following conditions: (A) 37°C, (B) 10 Gy, (C) 20 Gy, (D) GIHT, (E) GIHT + 10 Gy or (F) GIHT + 20 Gy in the presence or absence of GO, rGO and rGO-PEG. 24 hours post-treatment viability of the cells was investigated by flow cytometry . GIHT, graphene-induced hyperthermia; Gy, Gray; AxA5, annexin A5.

Supplementary figure 3.

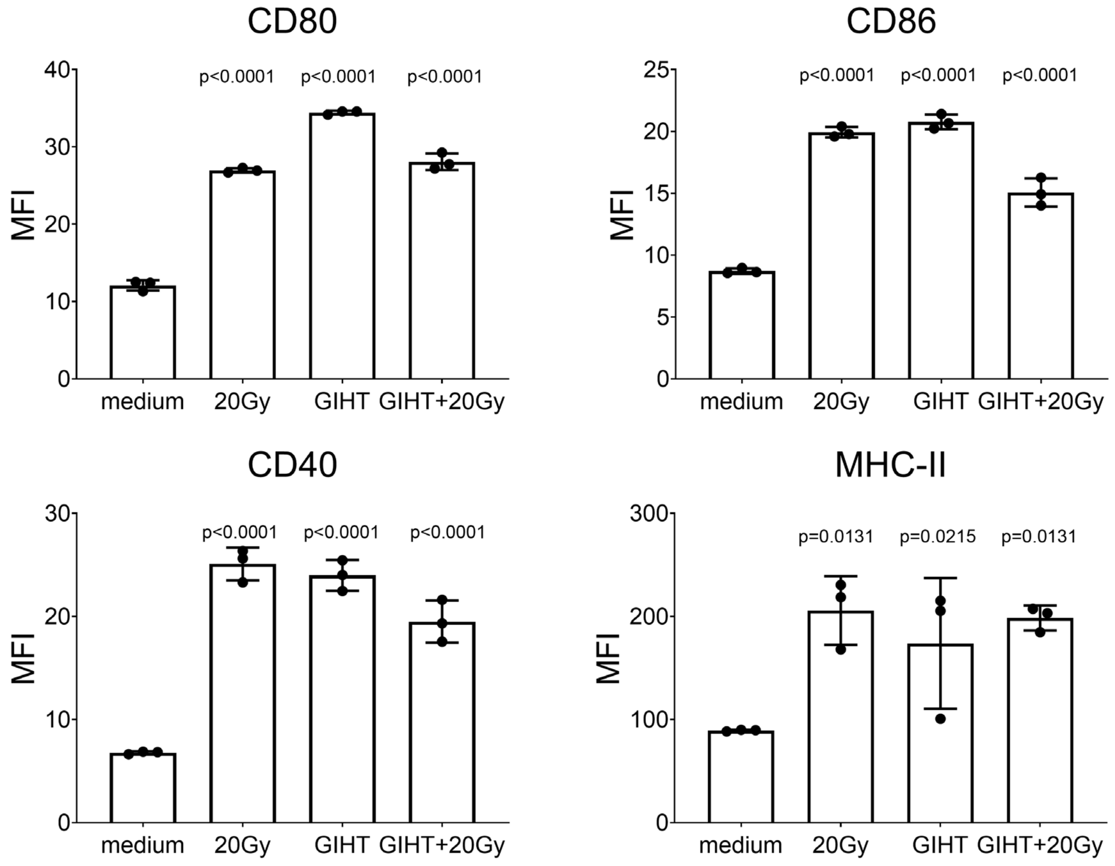

Expression of co-stimulatory molecules on dendritic cells. DCs were co-incubated for 24 hours with SN collected from B16F10 melanoma cells exposed to indicated treatments. One-way analysis of variances of five mice with Holm-Sidak´s multiple comparison test is shown. Values of $p < 0.05$ considered as significant are underlined. UNT, untreated; Gy, Gray; GIHT, graphene-induced hyperthermia.

Supplementary table 1

Multiple comparison test after Two way ANOVA of tumor volume (Figure 5C)

Within each day, compare treatments (simple effects within days)								
Number of families	5							
Number of comparisons per family	2							
Alpha	0.05							
**Uncorrected Fisher's LSD**	Predicted (LS) mean diff.	95.00% CI of diff.	Significant?	Summary	Individual P Value			
Day 0								
control vs. GIHT + 20 Gy	-3.411e-013	-682.0 to 682.0	No	ns	>0.9999			
control vs. 20 Gy	0.000	-747.1 to 747.1	No	ns	>0.9999			
Day 8								
control vs. GIHT + 20 Gy	36.94	-645.0 to 718.9	No	ns	0.9145			
control vs. 20 Gy	47.67	-699.4 to 794.7	No	ns	0.8994			
Day 12								
control vs. GIHT + 20 Gy	22.69	-659.3 to 704.7	No	ns	0.9474			
control vs. 20 Gy	206.0	-541.1 to 953.1	No	ns	0.5852			
Day 14								
control vs. GIHT + 20 Gy	294.4	-387.5 to 976.4	No	ns	0.3933			
control vs. 20 Gy	-382.2	-1129 to 364.8	No	ns	0.3121			
Day 16								
control vs. GIHT + 20 Gy	755.7	73.75 to 1438	Yes	*	**0.0303**			
control vs. 20 Gy	-1092	-1839 to -345.3	Yes	**	**0.0046**			
Test details	Predicted (LS) mean 1	Predicted (LS) mean 2	Predicted (LS) mean diff.	SE of diff.	N1	N2	t	DF
Day 0								
control vs. GIHT + 20 Gy	1.000	1.000	-3.411e-13	343.3	6	9	9.936e-016	90.00
control vs. 20 Gy	1.000	1.000	0.000	376.0	6	6	0.000	90.00
Day 8								
control vs. GIHT + 20 Gy	48.67	11.72	36.94	343.3	6	9	0.1076	90.00
control vs. 20 Gy	48.67	1.000	47.67	376.0	6	6	0.1268	90.00
Day 12								
control vs. GIHT + 20 Gy	311.3	288.6	22.69	343.3	6	9	0.06611	90.00
control vs. 20 Gy	311.3	105.3	206.0	376.0	6	6	0.5478	90.00
Day 14								
control vs. GIHT + 20 Gy	975.5	681.1	294.4	343.3	6	9	0.8578	90.00
control vs. 20 Gy	975.5	1358	-382.2	376.0	6	6	1.016	90.00
Day 16								
control vs. GIHT + 20 Gy	2089	1333	755.7	343.3	6	9	2.202	90.00
control vs. 20 Gy	2089	3181	-1092	376.0	6	6	2.905	90.00
